# Modulatory Effects of Vasoactive Intestinal Peptide on Intestinal Mucosal Immunity and Microbial Community of Weaned Piglets Challenged by an Enterotoxigenic *Escherichia coli* (K88)

**DOI:** 10.1371/journal.pone.0104183

**Published:** 2014-08-07

**Authors:** Chunlan Xu, Youming Wang, Rui Sun, Xiangjin Qiao, Xiaoya Shang, Weining Niu

**Affiliations:** 1 The Key Laboratory for Space Bioscience and Biotechnology, School of Life Sciences, Northwestern Polytechnical University, Xi’an, Shaanxi, China; 2 Institute of Feed Science, College of Animal Sciences, Zhejiang University, Hangzhou, PR China; Charité, Campus Benjamin Franklin, Germany

## Abstract

Toll-like receptors (TLRs) recognize microbial pathogens and trigger immune response, but their regulation by neuropeptide-vasoactive intestinal peptide (VIP) in weaned piglets infected by enterotoxigenic *Escherichia coli* (ETEC) K88 remains unexplored. Therefore, the study was conducted to investigate its role using a model of early weaned piglets infected by ETEC K88. Male Duroc×Landrace×Yorkshire piglets (n = 24) were randomly divided into control, ETEC K88, VIP, and ETEC K88+VIP groups. On the first three days, ETEC K88 and ETEC K88+VIP groups were orally administrated with ETEC K88, other two groups were given sterile medium. Then each piglet from VIP and ETEC K88+VIP group received 10 nmol VIP intraperitoneally (i.p.) once daily, on day four and six. On the seventh day, the piglets were sacrificed. The results indicated that administration of VIP improved the growth performance, reduced diarrhea incidence of ETEC K88 challenged pigs, and mitigated the histopathological changes of intestine. Serum levels of IL-2, IL-6, IL-12p40, IFN-γ and TNF-α in the ETEC K88+ VIP group were significantly reduced compared with those in the ETEC group. VIP significantly increased IL-4, IL-10, TGF-β and S-IgA production compared with the ETEC K88 group. Besides, VIP could inhibit the expression of TLR2, TLR4, MyD88, NF-κB p65 and the phosphorylation of IκB-α, p-ERK, p-JNK, and p-38 induced by ETEC K88. Moreover, VIP could upregulate the expression of occludin in the ileum mucosa compared with the ETEC K88 group. Colon and caecum content bacterial richness and diversity were lower for pigs in the ETEC group than the unchallenged groups. These results demonstrate that VIP is beneficial for the maturation of the intestinal mucosal immune system and elicited local immunomodulatory activities. The TLR2/4-MyD88 mediated NF-κB and MAPK signaling pathway may be critical to the mechanism underlying the modulatory effect of VIP on intestinal mucosal immune function and bacterial community.

## Introduction

Weaning is often stressful for piglets and accompanied by morphological, histological, microbial, and immunological changes along the digestive tract, which caused diarrhea and reduced growth [1], [2]. Thus, weaned piglets are often subjected to myriad of enteric diseases, and these diseases are the leading cause of mortality and economic losses in the swine industry. The intestine is the major site of digestion, nutrient absorption and hydro-mineral exchange homeostasis, harbouring a complex microbiota and a highly evolved mucosal immune system. The mucosal immune system fulfils two functions, to mount active responses against pathogens and to mount tolerance against harmless food and commensal bacterial antigens [3], [4]. Gut microbiota may play an important role in host health [5]. In the absence of the gut microbiota, normal immune development and function are impaired. The main challenge in a young animal is to obtain a balanced microbial population to prevent the establishment of pathogenic microorganisms [3]. Understanding the factors that influence the intestinal mucosal immunity and composition of the microbial community in the piglet infected by pathogens is crucial in regulating the intestinal immunity function and microflora, which will improve animal performance. Consequently, identification of the factors controlling the intestinal mucosal immunity, bacterial acquisition and community composition is of particular significance.

Under physiological and pathological conditions the enteric nervous system regulates intestinal mucosal function [6]. The small intestine possesses a net-work fiber that contains immunomodulating neuropeptides. VIP is an important signal molecule of the neuroendocrine-immune network [7], and a well characterized endogenous anti-inflammatory neuropeptide with therapeutic potential for a variety of immune disorders [8]. It is a member of the secretin-glucagon family and is involved in the modulation of numerous biological functions. It is known to affect the gastrointestinal, neuronal, and endocrine as well as the circulatory and immune systems [9]. A beneficial effect of VIP on experimental animal models of acute and chronic inflammation, such as acute pancreatitis [10], septic shock [11], arthritis [12], inflammatory bowel disease [13] and lipopolysaccharide (LPS)-induced acute inflammatory [14], has been demonstrated. Recently, VIP has been incorporated into the list of prospective immunotherapeutics for the treatment of inflammatory and autoimmune disorders. However, the possible protective effect of VIP achieved in the intestine mucosal immunity of piglets still remains obscure. Moreover, VIP displayed a direct antimicrobial activity against a variety of pathogens, including bacteria [15]. It was recently reported that VIP and its derivatives showed the strongest antimicrobial activities against *E.coli* strains that express complete O-antigen-containing LPS [16]. These antimicrobial activities add a further dimension to the immunomodulatory roles for VIP in the inflammatory and immune responses. Additionally, the recent discovery of Toll-like receptors (TLRs) has improved our understanding of the induction of both innate and adaptive immune responses against infection and injury. TLRs are implicated in protective immunity as well as in many inflammatory and autoimmune diseases; inhibitors of TLR signaling are being harnessed for a variety of therapeutic applications. Neuroendocrine mediators have been shown to play an important role in modulating both aspects of TLR regulation contributing to the endogenous control of homeostasis among the different players implicated in defense mechanisms [7]. However, the role of TLRs/nuclear factor-kappa-B (NF-κB) and (or) TLRs/mitogen-activated protein kinases (MAPK) in modulatory effects of VIP on intestinal mucosal immune function in early weaned piglets under infection is unclear. Moreover, in a preliminary in vitro study, VIP was shown to be effective against Enterotoxigenic *Escherichia coli* (ETEC), but this observation has not been confirmed in vivo.

Thus, in the present study, we used early weaned piglets infected by ETEC K88 as model to evaluate the morphologic alterations in the intestinal mucosa, and investigate the effect VIP on intestinal mucosal immunity and bacterial community, and the role of TLRs/myeloid differentiation factor 88 (MyD88)/NF-κB and (or) TLRs/MyD88/MAPK in modulatory effect of VIP on intestinal mucosal immune function under infection by ETEC K88. In addition, we investigated the involvement of paracellular pathway in the intestinal damage by evaluating the expression of the critical protein occludin. The information could provide valuable evidence for investigating the benefit effect of VIP on weaned piglet and explaining the mechanism of immune modulation by the porcine neuropeptide VIP.

## Materials and Methods

### Ethics Statement and experimental animals

This study was carried out in strict accordance with the recommendations in the Guide for the Care and Use of Laboratory Animals of the National Institutes of Health. The protocol was approved by the Committee on the Ethics of Animal Experiments of College of Animal Sciences, Zhejiang University (Permit Number: 2012072701) and the Committee on the Ethics of Animal Experiments of School of Life Sciences, Northwestern Polytechnical University (Permit Number: 12-015). All efforts were made to minimize suffering. Twenty four male Duroc×Landrace×Yorkshire piglets, 28 days of age and weighing 7.70±0.71 kg had been weaned at 21 days after birth. The feeding trial was carried out in the Swine Research and Teaching Farm at Zhejiang University. The disposal of experimental animals is strict comply with the management requirements of experimental animals.

### Bacterial strain and reagents

The ETEC strain used in this study was kindly donated by Professor Yizhen Wang of Zhejiang University. The enterotoxigenic *E.coli* was confirmed by Polymerase Chain Reaction (PCR) genotyping as genes expressing K88 fimbrial antigen and primarily cultured in Luria broth (LB) medium. Bacteria growing at 37°C in LB broth to log stationary phase (OD_600 nm_ of 0.8) was adjusted to a final concentration of 1×10^10^ CFU/ml before being used in the current experiment. Mainly Antibodies used in the experiment were as following: IκB-α (EPI), Ser^32/36^-phosphorylated IκB-α (CST), NF-κB p65 (Santa), p44/42 MAPK (ERK1/2) (Bioworld), p-p44/42 (p-ERK1/2) (Bioworld), JNK/SAPK (Santa), p-JNK/SAPK(Santa), p38 MAPK (EPI), p-p38 MAPK(CST), TLR2(EPI), TLR4(Santa), MyD88(Abcam), occludin and β**-**actin (Santa). Other reagents were obtained from Sigma Chemical Co. (St. Louis, MO, USA) unless otherwise mentioned.

### Establishment of the animal model

Twenty four 28-day male crossbred (Duroc×Landrace×Yorkshire) piglets weaned at 21 d were randomly divided into four groups of six piglets each: control, ETEC K88, ETEC K88+VIP, and VIP. Before infection, all the animals’ fecal samples were confirmed as being free of ETEC K88 by PCR. All piglets were housed in stainless steel pens (1.5×1.0 m) (three piglets per pen) with plastic-coated and wood-expanded floors at 25°C and under constant light with *ad libitum* access to feed and water. Moreover, ETEC K88 challenged groups and none-ETEC K88 challenged groups were housed in different unit with same environment conditions to prevent cross infection in the process of experiment. All treatments received the same basal diets. Diets were formulated to meet requirements for all nutrients and did not contain any antibiotics or medicine. After an acclimation period of 7 days, twelve piglets from ETEC K88 and ETEC K88+VIP groups were infected orally with 1×10^10^ CFU/ml of ETEC K88 [17], [18], whereas the control and VIP groups received sterile medium orally on the first 3 days. Then each piglet from VIP and ETEC K88+VIP groups were given 10 nmol VIP (GL Biochem (shanghai) Ltd, China) intraperitoneally (i.p.) once daily, on day four and six based on previous reports [19], [20] and the preliminary experiment in our Lab. Following the same protocol, piglets from control and ETEC K88 groups were given the same volumes of normal saline.

### Animal observation and sample collection

All the infected piglets were fecal-culture positive for ETEC K88 and developed similar clinical signs of gastrointestinal disease, including increased rectal temperature, diarrhea and lethargy. The Average daily gain (ADG), Average daily feed intake (ADFI), and Body weight gain efficiency (G:F) of each pig were monitored throughout the experimental period. The number of pigs with diarrhea was recorded daily, and the diarrhea ratio was calculated according to the following equation: diarrhea ratio = total number of pigs with diarrhea/(total number of experimental pigs×trail days)×100. On the sixth day after the first infection, all pigs were anesthetized with Zoletil (20 mg/kg, i.m.) and blood samples were drawn in collection tubes by venipuncture of the anterior vena cava of pigs. The pigs were euthanized with an overdose of the anesthetic. Serum was obtained after centrifugation at 3000 g for 15 min at 4°C and stored at −80°C until further use. Tissue sampling included collection of duodenal (5 cm distal of the pyloric sphincter), jejuna (35 cm distal of the pyloric sphincter), and ileum segments (10 cm proximal to the ileocecal junction). One 4 cm long piece segment from each region was divided into two pieces. One piece was fixed with 4% paraformaldehyde for histological analysis. Caecum and distal colon content were collected in sterile sample bags, snap-frozen in liquid nitrogen and stored at −80°C until used for microbial DNA extraction. After collection of intestinal contents, mucosal tissue was gently scraped from the other piece using a sterile scalpel, and then mixture of mucosal scrapings was flash frozen in liquid nitrogen and stored at −80°C for protein isolation.

### Histological examination for the intestinal morphology

Partial intestine tissues (duodenum, jejuna and ileum) were fixed in 4% paraformaldehyde for 24 h, and then embedded in paraffin wax. Sections of 5 µm were cut and stained with hematoxylin and eosin (H&E). An independent pathologist blinded to the experimental group of the samples performed the histological analysis. Images were captured using a high-resolution Samsung camera coupled to a light Nikon E200 microscope and subsequently analyzed using AxioVision-Rel software (Zeiss). Total mucosal thickness (TMT), villous height (VH), and crypt depth (CD) were evaluated. The VH: CD (VCR) was calculated. Each variable was measured three times for all three portions of the intestine, so the final value of a given variable for one specimen is the mean of these nine measurements.

### Enzyme linked-immuno sorbent assay (ELISA) for detection of serum levels of cytokines and Secretory IgA (S-IgA)

Piglets serum was collected as described previously. The serum levels of interleukin-2 (IL-2), interleukin-4 (IL-4), interleukin-6 (IL-6), interleukin-10 (IL-10), interleukin-12 p40 (IL-12 p40), tumor necrosis factor α (TNF-α), transforming growth factor β (TGF-β), interferon γ (IFN-γ) and S-IgA were measured using commercially available ELISA kits (R&D, USA) in strict accordance with the manufacturer’s instructions. The color intensity was read using optical density (OD) at 450 nm with a tunable microplate reader (VersaMax, Molecular Devices, CA, USA) and the concentration of cytokine calculated from a standard curve.

### Western blot for detection of TLRs, MAPK and NF-κB signaling molecules and occludin expression

Protein was isolated from 200 mg of ileal mucosal tissue using a total protein extraction kit and cytoplasmic and nuclear protein extraction kit (Beyotime Biotechnology, Haimen, China) according to the manufacturer’s instructions. The BCA method was used to measure protein concentrations. Cytosolic occludin, IκB-α, p-IκB-α, p44/p42 (ERK1/2), p-p44/p42, p38, p-p38, JNK/SAPK, p-JNK/SAPK, TLR4, TLR2, MyD88, nuclear NF-κB p65 proteins and β-actin were detected. Cytosolic or nuclear proteins (35 µg) were loaded into 10% sodium dodecyl sulfate (SDS)-polyacrilamide gel electrophoresis (PAGE) and transferred to a 0.45 µm-pore polyvinylidene difluoride membrane (PVDF; Immuno-Blot, BioRad). The membranes were then incubated with blocking solution (150 mM NaCl, 20 mM Tris-HCl, 0.1% Tween 20, 5% skim milk, pH 7.4) for 1 h at room temperature (RT). After the blocking reaction, membranes were incubated with first antibodies against occludin (1∶800), IκB-α (1∶1000), p- IκB-α (1∶1000), p44/p42 (ERK1/2) (1∶1000), p-p44/p42 (1∶1000), p38 (1∶1000), p-p38 (1∶1000), JNK/SAPK (1∶500), p-JNK/SAPK (1∶500), p65 (1∶1000), TLR4 (1∶500), TLR2 (1∶1000), MyD88 (1∶500) and β-actin (1∶1000) for 90 min at RT, and then washed thrice with TBS-T (1% Tween 20 in Tris buffered saline) followed by incubation with secondary antibody-horseradish peroxidase-conjugated goat anti-rabbit IgG (Santa Cruz Biotechnology, CA, USA) for 1 h at RT. An ECL agent was added for chemiluminescence imaging. The images were collected using the Gel EQ system (Bio-Rad, Inc.), and the built-in software was used to analyze the gray values of the bands. The relative expression levels of the proteins were expressed as the gray value of the target band over the gray value of β-actin in the same sample. Each sample had 3 replicates.

### Composition and diversity of bacterial community through 454 pyrosequencing analysis

Before DNA extraction, equal masses of sub-samples (caecum or distal colon content) collected from each pigs in the same treatment group were pooled together and homogenized in a sterile Stomacher (Seward Laboratory, London, UK) at 4°C. Genomic DNA in caecum and colon contents was extracted using DNA Kit (Omega Bio-Tek) according to the manufacture’s protocol with slight modification, then identified by 1% agarose gel electrophoresis. According to the specific sequence region (533R-27F) in the 16S rRNA gene that covering the V1–V3 region, the bar-coded primers 27F and 533R containing the A and B sequencing adaptors were synthesized and used to amplify this region. The forward primer (B-27F) was 5′-*CCTATCCCCTGTGTGCCTTGGCAGTCTCAG*AGAGTTTGATCCTGGCTCAG-3′, where the sequence of the B adaptor is shown in italics and underlined. The reverse primer (A-533R) was 5′-*CCATCTCATCCCTGCGTGTCTCCGACTCAG* NNNNNNNNNNTTACCGCGGCTGCTGGCAC-3′, where the sequence of the A adaptor is shown in italics and underlined and the Ns represent an eight-base sample specific barcode sequence. The identified DNA was subjected to polymerase chain reaction (PCR) using TranStartFastpfu DNA Polymerase (MBI. Fermentas, USA) in a 20 µL volume containing 5 mM each of the primer, 10 ng of template DNA, and 5×FastPfu Buffer, 1 U of FastPfu DNA Polymerase. PCR was performed in a thermocycler (Gene Amp PCR System 9700, ABI, USA). The PCR profile included denaturation at 95°C for 2 min, followed by 25 cycles of denaturation at 95°C for 30 s, annealing at 55°C for 30 s, and extension at 72°C for 30 s, and a final extension at 72°C for 5 min. Triplicate PCR products of the same sample were mixed, and then detected by 2% agarose gels electrophoresis containing ethidium bromide. PCR products were recycled and purified with a AxyPreDNA gel extraction kit (Axygen, China) according to the manufacture’s instruction. The recycled PCR products were visualized on agarose gels. Furthermore, the PCR products were quantitatively determined using QuantiFluor-ST Fluoremeter (Promega, USA) and PicoGreen dsDNA Quantitation Reagent (Invitrogen, Germany) Following quantitation, the amplification from each reaction mixture were pooled in equimolar ratios based on concentration and subjected to emulsion PCR (emPCR) using RocheGS FLX Titanium emPCR kits to generate amplification libraries. Amplification pyrosequencing was performed from the A-end using a 454/Roche A sequencing primer kit on a Roche Genome Sequencer GS FLX Titanium platform at Majorbio Bio-Pharm Technology Co., Ltd., Shanghai, China.

### Statistical analysis

Statistical analyses were performed using SPSS 18.0 (Chicago, IL, USA). Independent sample t-test was adopted to determine significant differences, in which the data were expressed as mean ± standard error of mean (SEM), and differences were considered significant at *p*<0.05. The pyrosequencing data were subjected to bioinformatic analysis. Prior to analyze, the original pyrosequencing data must be filtered and optimized to obtain the valid and trimed sequences through Seqcln and Mothur(http://sourceforge.net/projects/seqclean/ & http://www.mothur.org/wiki/Main_Page). Then, these trimed sequences were analyzed from two aspects: operational taxonomic units (OTUs) cluster (97% similarity) and taxonomy which mainly performed on Mothur (http://www.mothur.org) and compared with the Bacterial SILVA database (http://www.arb-silva.de/), and by methods of kmer searching (http://www.mothur.org/wiki/Align.seqs) and UCHIME (http://drive5.com/uchime). Rarefaction analysis and Good’s coverage for the nine libraries were determined. Community figure was generated using R tools according to the data from document “tax.phylum.xls”. Heatmap figure were generated using Vegan-package (distance measure: Bray-Curtis; cluster analysis: complete).

## Results

### Growth performance and diarrhea ratio

The effect of administration with VIP on growth performance and diarrhea ratio are presented in Table 1. The ADG (*P*<0.001), ADFI (*P*<0.001), and G:F (*P*<0.01) of piglets in ETEC K88+ VIP group was higher than those of piglets in ETEC K88 group. The pigs in the VIP group presented a significantly higher ADG (*P*<0.001), ADFI (*P*<0.001), and G:F (*P*<0.001) compared with the pigs in the control group. Administration with VIP effectively alleviated the incidence of diarrhea in ETEC K88 challenged piglets. None of the piglets in group control and VIP showed any signs of diarrhea throughout the experiment.

**Table 1:** Effect of VIP on growth performance and diarrhea incidence of piglets challenged with ETEC K88.

Item	Control	ETEC K88	ETEC K88+ VIP	VIP
ADG, g/d	200.00±4.24	98.33±4.37^###^	136.7±5.34***	251.7±5.08^###^
ADFI, g/d	284.38±4.88	189.08±5.84^###^	238.65±4.17***	315.67±2.36^###^
G:F	0.70±0.00	0.52±0.01^###^	0.57±0.01**	0.80±0.01^###^
Diarrhea incidence, %	0	32.58±2.06^###^	12.72±0.95***	0

Note: ^###^ *P*<0.001 compared to Control; **P<0.01, ***P<0.001 compared to ETEC K88. Values are expressed as mean ± SEM. ADG, ADFI, G:F represents average daily gain, average daily feed intake, and feed conversion efficiency (G:F), respectively.

### Intestinal morphology

**Figure 1:**
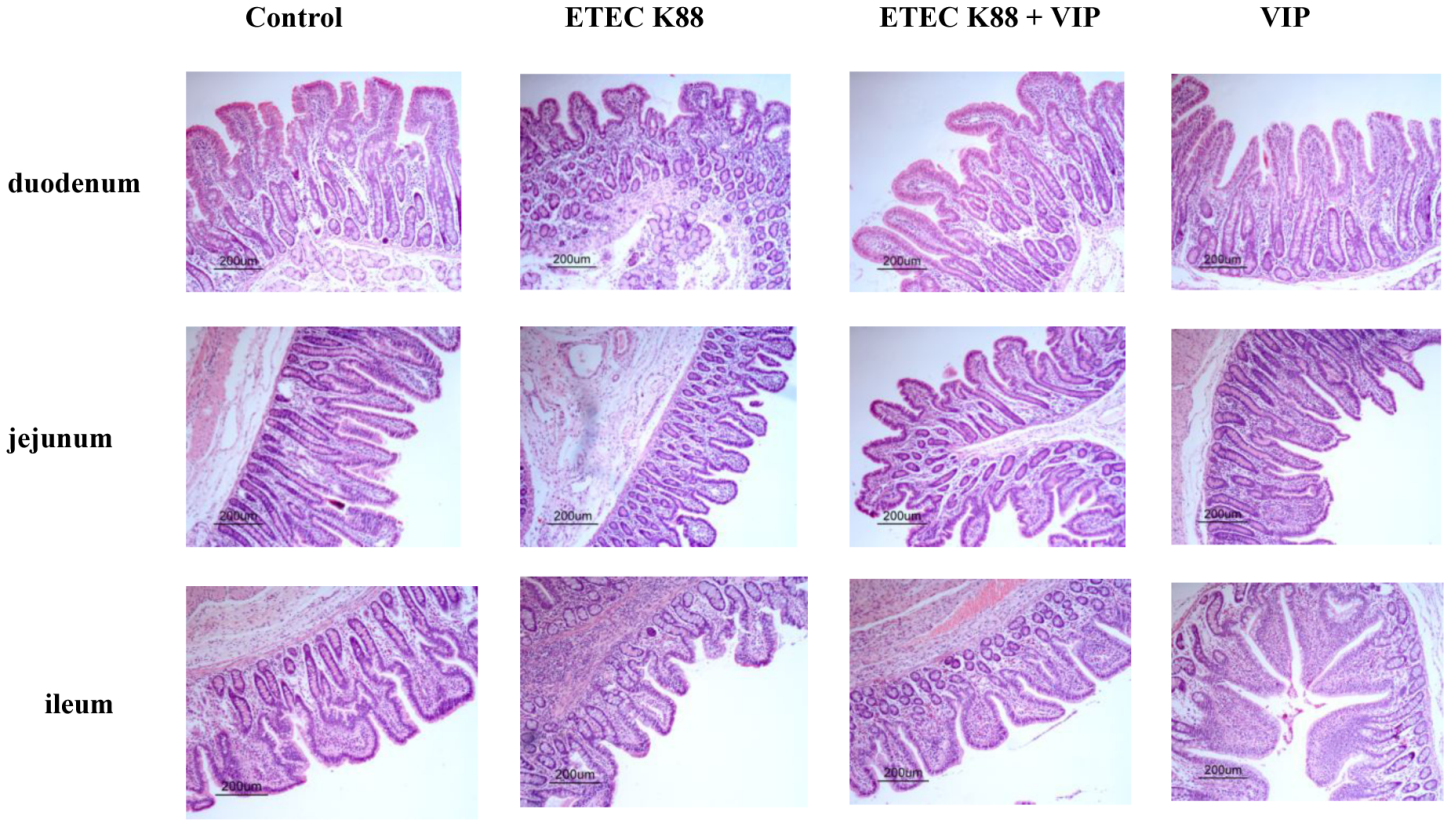
Influence of VIP on morphology of intestine in piglets infected by ETEC K88 (H&E).

As shown in Figure 1 and Table 2, the histological analyses of the intestine showed that a significant decrease in VH (duodenum: *P*<0.01; jejunum: *P*<0.01; ileum: *P*<0.05), CD (duodenum: *P*<0.001) and TMT (duodenum: *P*<0.001; jejunum: *P*<0.001) in the ETEC K88 group when compared with the control group (). No differences (*P*>0.05) among treatments were observed in the VCR values. Compared with the ETEC K88 group, the reduction in TMT (duodenum: *P*<0.001; jejunum: *P*<0.001; ileum: *P*<0.05), VH (duodenum: *P*<0.001; jejunum: *P*<0.001; ileum: *P*<0.01), and CD (duodenum: *P*<0.001; jejunum: *P*<0.001) were lower in the ETEC K88+ VIP group. The intestinal morphology (VH, CD, and VCR) of piglets in the control group did not differ from those of piglets receiving VIP (*P*>0.05). These results indicated that VIP may have the positive regulation function on the intestinal tract, which may be related to the decline of diarrhea cause by ETEC K88.

**Table 2:** Effect of VIP on morphology of the intestines in weaned piglets infected by ETEC K88.

Items	Groups			
	Control	ETEC K88	ETEC K88+VIP	VIP
Duodenum				
Villous height (VH), µm	327.80±11.78	232.10±25.83^##^	414.10±23.16***	355.50±12.22
Crypt depth (CD), µm	270.20±11.73	173.20±16.95^###^	305.90±13.24***	271.50±16.67
Total mucosal thickness, µm	640.70±7.25	455.20±9.91^###^	691.10±27.96***	631.80±17.04
VH:CD	1.23±0.08	1.37±0.15	1.37±0.10	1.33±0.06
Jejunum				
Villous height (VH), µm	281.10±12.13	215.30±10.99^##^	314.30±18.48***	307.60±12.96
Crypt depth (CD), µm	172.70±4.72	145.80±7.16	203.30±3.31***	181.31±15.30
Total mucosal thickness, µm	596.70±16.49	396.9±11.72^###^	572.60±10.12***	607.70±18.90
VH:CD	1.64±0.09	1.51±0.14	1.54±0.08	1.70±0.05
Ileum				
Villous height (VH), µm	276.10±12.56	214.6±18.15^#^	288.10±15.32**	285.10±12.94
Crypt depth (CD), µm	159.40±5.48	140.40±8.29	169.40±6.57	168.20±11.34
Total mucosal thickness, µm	424.80±5.56	400.00±16.01	450.00±17.29*	476.80±9.81^#^
VH:CD	1.75±0.11	1.52±0.06	1.71±0.10	1.75±0.14

Note: ^#^ *P*<0.05, ^##^ *P*<0.01, ^###^ *P*<0.001 compared to Control; *P<0.05, **P<0.01, ***P<0.001 compared to ETEC K88. Values are expressed as mean ± SEM (n = 6, each group).

### Serum levels of cytokines and S-IgA

**Figure 2:**
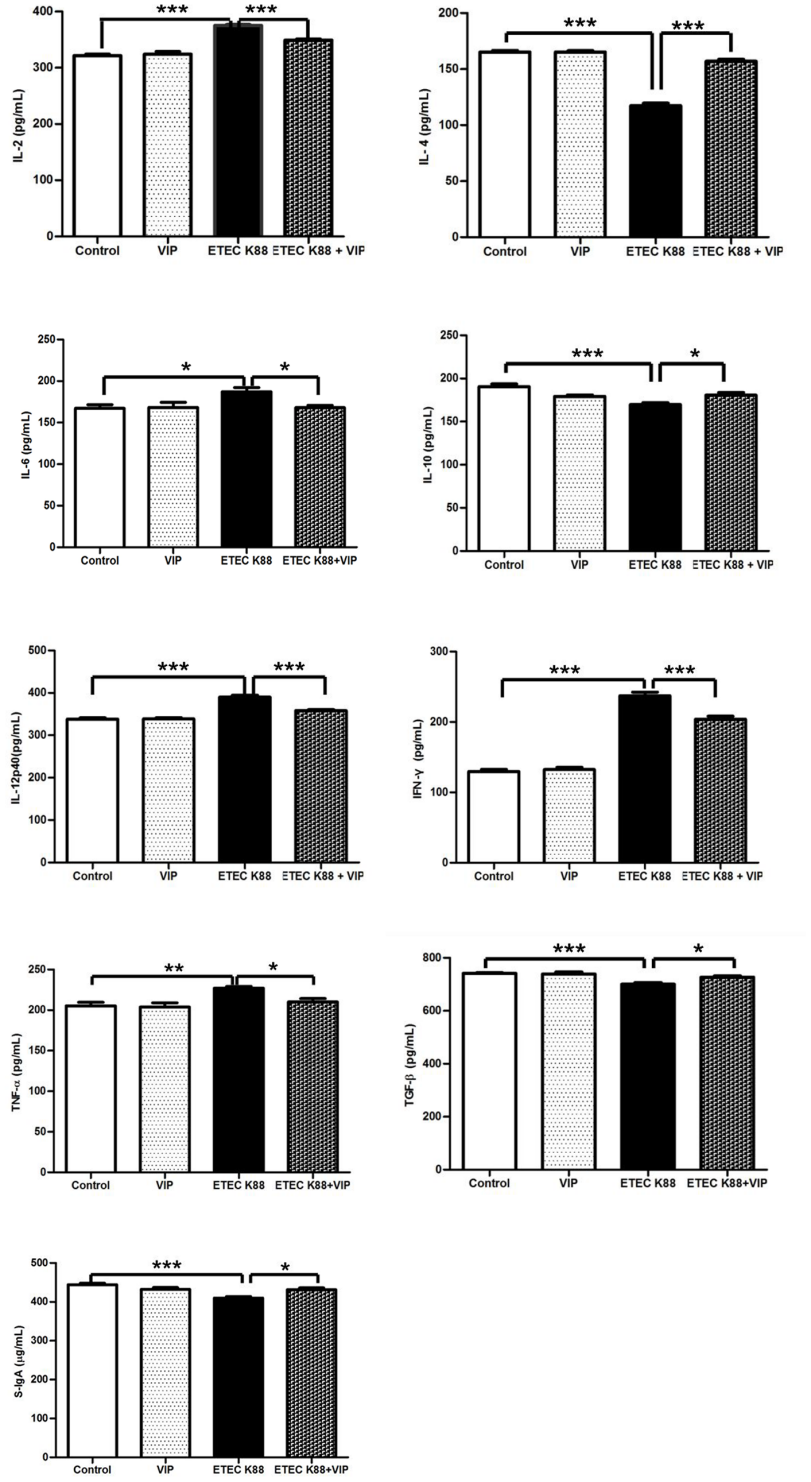
Effect of VIP on expression of immune-related molecules in serum from piglets infected with ETEC K88. Each results is the mean (n = 6)±S.E.M. of cytokines levels determined in triplication. *P<0.05, **P<0.01, ***P<0.001.

As shown in Figure 2, the serum concentrations of IL-2 (*P*<0.001), IL-6 (*P*<0.05), IL-12p40 (*P*<0.001), IFN-γ (*P*<0.001) and TNF-α (*P*<0.01) in the ETEC K88 group were significantly higher than those in the control group, suggesting ETEC K88 induced inflammatory response. The concentrations of IL-4 (*P*<0.001), IL-10 (*P*<0.001), TGF-β (*P*<0.001) and S-IgA (*P*<0.001) in the serum of piglets from the ETEC K88 group were significantly lower than the concentrations in those from the control group. However, VIP-treatment significantly reduced the serum levels of IL-2 (*P*<0.001), IL-6 (*P*<0.05), IL-12p40 (*P*<0.001), IFN-γ (*P*<0.001) and TNF-α (*P*<0.05), increased the serum levels of IL-4 (*P*<0.001), IL-10 (*p*<0.05), TGF-β (*p*<0.05) and S-IgA (*p*<0.05) compared with the ETEC K88 group. These results suggested that VIP-treatment reversed ETEC K88-induced increase of inflammatory mediators.

### TLRs, MAPK, NF-κB and occludin protein expression

**Figure 3:**
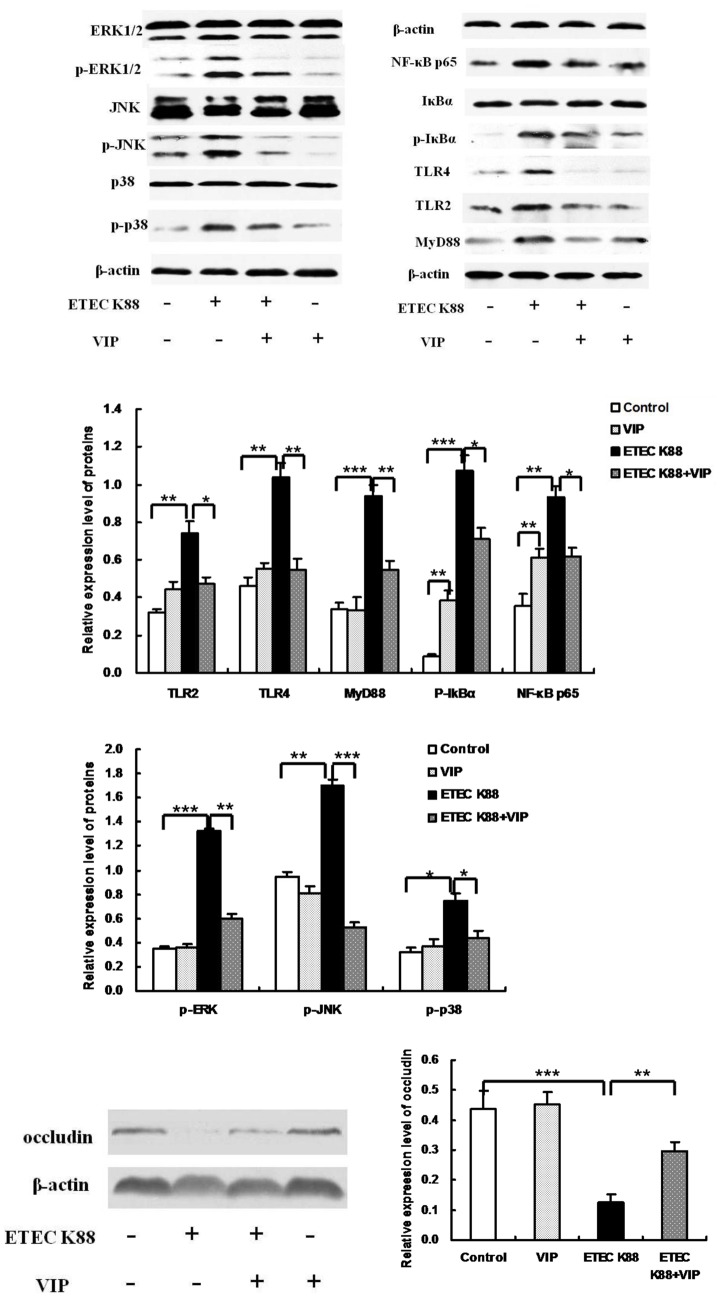
Effect of VIP on degradation and phosphorylation of IκBα, ERK1/2 (p44/p42) MAPK, p38 MAPK, and JNK/SAPK, and expression of TLRs, NF-κB p65, MyD88 and occludin in the ileum. Cytosolic occludin, IκB-α, p-IκB-α, p44/p42 (ERK1/2), p-p44/p42, p38, p-p38, JNK/SAPK, p-JNK/SAPK, p65, TLR4, TLR2, MyD88, nuclear NF-κB p65 proteins and actin were detected by western blot analysis. Each results is the mean (n = 6)±S.E.M. *P<0.05, **P<0.01, ***P<0.001.

Toll-like receptors (TLRs) are a family of pattern-recognition receptors that play a key role in the innate immune system. Western blot (Figure 3) analysis showed that the expression levels of TLR2 and TLR4 in the ETEC K88+VIP group were lower than that in the ETEC K88 group, and MyD88 levels in the ETEC K88+VIP group were lower than that in group ETEC K88. Activation of NF-κB and MAPKs, particularly the stimulation of ERK subgroup, has been demonstrated as the critical signals to trigger the cytokine production from immune-responsive cells. So we detected the phosphorylation of MAPKs and NF-κB pathways proteins by western blotting. Reduce phosphorylation of IκB-α was observed in the ETEC K88+VIP group. The expression levels of NF-κB p65 in the ETEC K88 group were higher than that in the control group. Administration of VIP significantly inhibited the expression of NF-κB p65, the phosphorylation of IκB-α compared with the ETEC K88 group. Since the activation of MAPK requires phosphorylation of threonine or tyrosine residues, antibodies against ERK, JNK, and p38 were used and their phospho-forms detected using western blot. The data show administration of VIP significantly inhibited the phosphorylation of p-38, ERK and JNK compared with the ETEC K88 group. In addition, we investigated the involvement of paracellular pathway in the intestinal damage by evaluating the expression of this critical protein occludin. As shown in Figure 3, group ETEC K88 showed significantly decreased expression of occludin compared to the control group. VIP administration rescued ETEC K88 induced reduction of occludin, as shown by increased occludin protein in the ETEC K88+VIP group.

### Bacterial composition and diversity in the caecum and colon content

**Figure 4:**
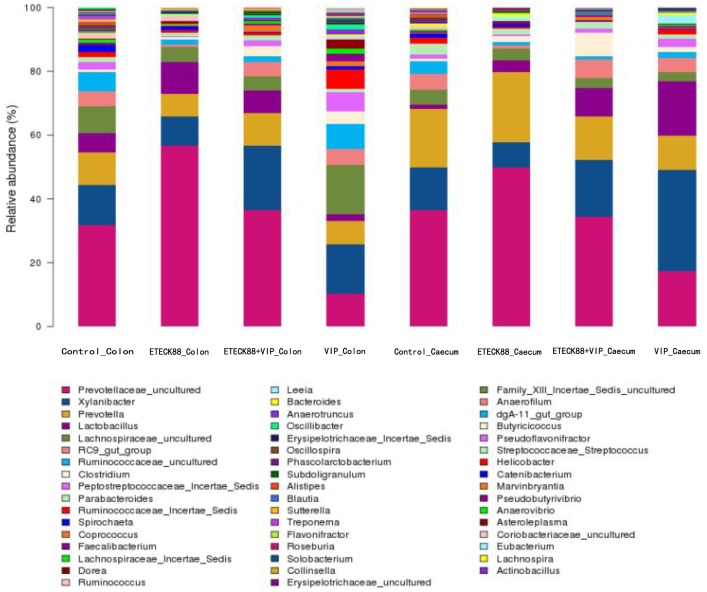
Bacterial composition of the different communities. Relative read abundance of different bacterial genus within the different communities. Sequences that could not be classified into any known group were assigned as “unclassified bacteria”. The ETEC K88-challenged pigs and unchallenged pigs with same breed and age, and similar weight were not from the same litter and were assigned to the four treatments in a randomized complete block design. All pigs were housed in stainless steel pens (three piglets per pen) in the same unit.

**Figure 5:**
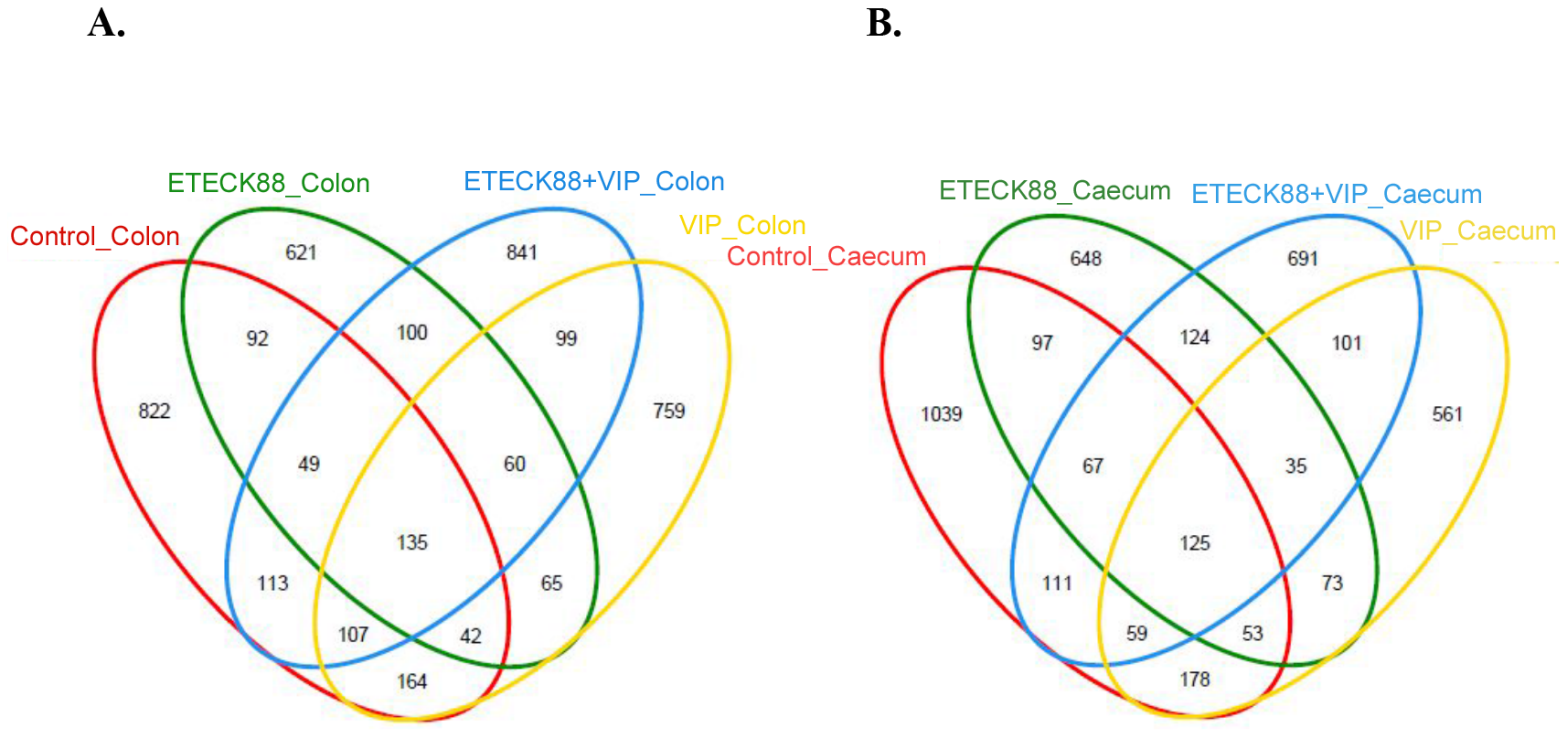
Shared OUT analysis of the different libraries. Venne diagram showing the unique and shared OTUs (3% distance level) in the different libraries (A) for the colon libraries from different treatments, and (B) for the caecum libraries from different treatments.

**Figure 6:**
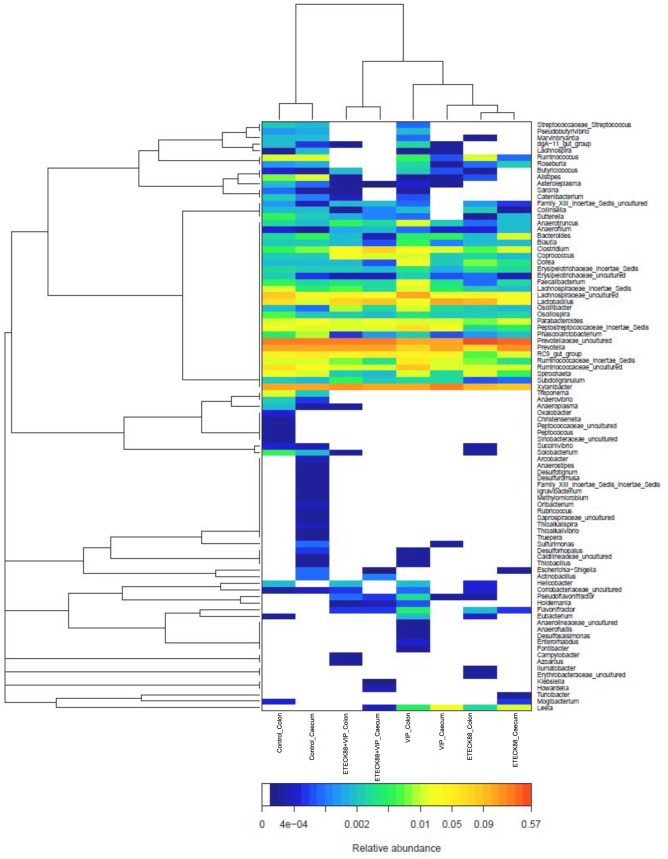
Bacterial distribution among the eight samples. Double hierarchical dendrogram showing the bacterial distribution among the samples. The bacterial phylogenetic tree was calculated using the neighbor-joining method and the relationship among samples was determined by Bray distance and the complete clustering method. The heatmap plot depicts the relative percentage of each bacterial family are depicted by color intensity with the legend indicated at the bottom of the figure. Clusters based on the distance of the eight samples along the X-axis and the bacterial families along the Y-axis are indicated in the upper and left of the figure, respectively.

A total of 104,799 valid reads and 6, 009 operational taxonomic units (OTUs) were obtained from the samples through 454 pyrosequencing analysis. 53 reads and 49 OTUs were eukaryotes and were therefore excluded in the subsequent analysis. Good’s coverage estimations revealed that 89.7% to 98% of the species were obtained in all of the samples. All sequences were classified from phylum to genus according to the program Mothur using the default setting. 14 different phyla and 50 different genuses were identified from these samples. The eight libraries showed very dissimilar 16S rRNA profiles even in phylum level distributions (Figure 4). The most abundant OTUs associated with the colon digesta from the VIP treatment alone group were *Xylanibacter* (15.56%) and *Lachnospiraceae* (15.28%). The OUT composition and abundance was relatively similar between the colon and caecum content in the same group, two ETEC K88 infection groups, and the control and VIP treatment alone group (data not shown). The bacterial species in the colon and caecum digesta libraries were further investigated for the presence of a core gut microbiota. Figure 5 and Table 3 showed that the colon and caecum content libraries from each experimental group have 135 and 125 OTUs in common, respectively. *Bacteroidetes* and Firmicutes included 112 (colon content, 82.96% in proportion) and 101 (caecum content, 80.80% in proportion) of the shared OTUs, and 30013 (colon content) and 28852 (caecum content) shared reads. Within these two phyla, *Bacteroidia*, *Bacilli*, *Clostridia* and *Erysipelotrichi* represented the most abundant classes common to the eight libraries. For *Actinobacteria*, *Proteobacteria*, *Tenericutes* and *Spirochaetes*, OTUs common was very little, and they tended to be low in abundance. Hierarchically clustered heatmap analysis based on the bacterial community profiles at family level disclosed that the samples from the same group grouped together firstly except for VIP treatment alone group, and they then clustered with samples from VIP treatment alone, ETEC K88 plus VIP and control groups in order (Figure 6). In addition, the significant differences analysis between groups showed that the bacterial composition in the ETEC K88 infected groups was significantly different from the control groups, and administration with VIP in the weaned piglets challenged with ETEC K88 significantly changed the bacterial composition and community compared with the ETEC K88 infection group. The different bacterial at the genus level between each groups were shown in Table 4 and 5.

**Table 3:** Shared phyla among the colon content and caecum content libraries*.

*Phylum*		Shared reads (colon content)					Shared reads (caecum content)			
	*Shared OTUs*	Control	ETEC K88	ETEC K88+VIP	VIP	*Shared OTUs*	Control	ETEC K88	ETEC K88+VIP	VIP
*Actinobacteria*	1	7	15	4	14	1	19	1	5	3
***Bacteroidetes***	68	3762	5408	5789	3093	62	6773	5981	5871	4610
***Firmicutes***	44	3437	1605	2257	4662	39	3297	1342	2161	2114
*Proteobacteria*	3	43	24	18	45	3	55	76	14	134
*Spirochaetes*	4	139	76	14	56	3	90	38	8	17
*Tenericutes*	2	413	318	12	246	2	215	207	21	71
unclassified	13	4	8	10	8	15	10	4	7	2
Total shared sequences	135	7801	7446	8094	8116	125	10449	7645	8080	6949
Total reads		7844	7485	8138	8165		10515	7696	8106	6975
Shared reads/Total reads (%)		99.45	99.48	99.46	99.4		99.37	99.34	99.68	99.63

*The phyla in bold letters represent core gut microbiota.

**Table 4:** Differentially Abundant Features of Colon Contents*.

Control vs. ETEC K88	ETEC K88 vs. ETEC K88+VIP	Control vs. VIP
***Alistipes***	***Anaerotruncus***	***Alistipes***
***Anaerotruncus***	***Butyricicoccus***	*Anaeroplasma*
*Anaerovibrio*	***Clostridium***	***Anaerotruncus***
***Catenibacterium***	***Collinsella***	*Anaerovibrio*
***Coprococcus***	***Coprococcus***	***Blautia***
***Dorea***	***Dorea***	***Butyricicoccus***
***Faecalibacterium***	***Erysipelotrichaceae_Incertae_Sedis***	***Catenibacterium***
*Flavonifractor*	***Faecalibacterium***	***Clostridium***
***Lachnospiraceae_Incertae_Sedis***	***Flavonifractor***	***Coprococcus***
***Lachnospiraceae_uncultured***	***Helicobacter***	***Dorea***
***Lactobacillus***	***Lachnospiraceae_Incertae_Sedis***	***Eubacterium***
*Leeia*	***Lactobacillus***	***Faecalibacterium***
***No_Rank***	*Leeia*	*Flavonifractor*
***Oscillospira***	***No_Rank***	***Lachnospiraceae_Incertae_Sedis***
***Peptostreptococcaceae_Incertae_Sedis***	***Oscillibacter***	***Lachnospiraceae_uncultured***
***Phascolarctobacterium***	***Parabacteroides***	***Lactobacillus***
***Prevotellaceae_uncultured***	***Peptostreptococcaceae_Incertae_Sedis***	*Leeia*
***Prevotella***	***Prevotellaceae_uncultured***	***No_Rank***
***RC9_gut_group***	***Prevotella***	***Oscillibacter***
***Ruminococcaceae_Incertae_Sedis***	***RC9_gut_group***	***Peptostreptococcaceae_Incertae_Sedis***
***Ruminococcaceae_uncultured***	*Ruminococcus*	***Prevotellaceae_uncultured***
***Solobacterium***	***Spirochaeta***	***Prevotella***
*Streptococcaceae_Streptococcus*	***Subdoligranulum***	*Pseudoflavonifractor*
***Sutterella***	***Xylanibacter***	***RC9_gut_group***
*Treponema*		***Roseburia***
*dgA-11_gut_group*		***Ruminococcaceae_Incertae_Sedis***
		***Ruminococcaceae_uncultured***
		***Ruminococcus***
		*Solobacterium*
		***Spirochaeta***
		***Streptococcaceae_Streptococcus***
		***Sutterella***
		Treponema
		**Xylanibacter**

*Bacterial community comparison between the groups in the level of genus; The genus listed in the table are significant between the groups. Among of them, shared genus of bacteria between groups is shown in italics bold.

**Table 5:** Differentially Abundant Features of Caecum Contents*.

Control vs. ETEC K88	ETEC K88 vs. ETEC K88+VIP	Control vs. VIP
*Alistipes*	***Anaerotruncus***	***Alistipes***
***Anaerofilum***	***Bacteroides***	***Blautia***
***Bacteroides***	***Clostridium***	***Clostridium***
***Clostridium***	***Coprococcus***	*Collinsella*
***Dorea***	***Dorea***	***Lachnospiraceae_uncultured***
***Erysipelotrichaceae_Incertae_Sedis***	***Family_XIII_Incertae_Sedis_uncultured***	***Lactobacillus***
***Lachnospiraceae_Incertae_Sedis***	***Lachnospiraceae_Incertae_Sedis***	*Leeia*
*Lachnospira*	***Lactobacillus***	*Marvinbryantia*
***Lactobacillus***	***Leeia***	*No_Rank*
*Leeia*	***No_Rank***	***Oscillibacter***
*Marvinbryantia*	***Oscillibacter***	***Parabacteroides***
***No_Rank***	***Peptostreptococcaceae_Incertae_Sedis***	***Peptostreptococcaceae_Incertae_Sedis***
***Oscillospira***	***Prevotellaceae_uncultured***	***Phascolarctobacterium***
***Parabacteroides***	***Prevotella***	***Prevotellaceae_uncultured***
***Peptostreptococcaceae_Incertae_Sedis***	***RC9_gut_group***	***Prevotella***
***Phascolarctobacterium***	*Roseburia*	***Ruminococcaceae_uncultured***
***Prevotellaceae_uncultured***	***Spirochaeta***	***Ruminococcus***
***Prevotella***	***Subdoligranulum***	*Solobacterium*
*Pseudobutyrivibrio*	***Xylanibacter***	***Spirochaeta***
***RC9_gut_group***		*Streptococcaceae_Streptococcus*
***Roseburia***		***Subdoligranulum***
***Ruminococcaceae_Incertae_Sedis***		*Sutterella*
***Ruminococcaceae_uncultured***		*Treponema*
***Ruminococcus***		***Xylanibacter***
*Solobacterium*		
*Streptococcaceae_Streptococcus*		
*Treponema*		
***Xylanibacter***		

*Bacterial community comparison between the groups in the level of genus; The genus listed in the table are significant between the groups. Among of them, shared genus of bacteria between groups is shown in italics bold.

## Discussion

Diarrhea in neonatal and early-weaned piglets due to ETEC is an important problem in the pig farming industry. The VIP is a well-characterized endogenous anti-inflammatory neuropeptide with therapeutic potential for a variety of immune disorders. The experiment was aimed to investigate whether exogenous VIP would protect against ETEC infection of piglets fed a diet without supplemental antibiotics. The current results indicated that the growth performance was impaired, and incidence of diarrhea was increased in piglets challenged by enterotoxigenic ETEC K88, which was in consistent with previous observation [17], [18], [21]. ETEC K88 is a major cause of diarrhea and death in neonatal and weaned pigs [22]. ETEC K88 not only colonize in the small intestine, but also release enterotoxins to stimulate the epithelial cells to secrete fluid into the lumen of the gut to cause diarrhea [23]. However, treatment with VIP significantly improved the ETEC K88 challenge-caused signs as indicated by attenuated growth depression and decreased diarrhea incidence in piglets.

The intestine of weaned piglet is very susceptible to pathogenic microorganisms, and severe changes occur in the intestinal epithelium after this insult. Furthermore, reductions in VH have been associated with poor growth performance and increased incidences of scouring in pigs challenged with ETEC [24]. A feature of ETEC infection is effacing of the intestinal mucosa, which often leads to shorter villous and deeper crypts [2]. The morphometric evaluation showed that a reduction in mucosal thickness in the ETEC K88 group. This reduction was mainly attributable to the loss in VH, with a relative sparing of the CD. However, longer VH in ETEC K88 infected-piglets receiving VIP than in ETEC K88 infected-piglets alone was observed in the study. Longer VH are often used as an indicator of an increased absorptive capacity of the small intestinal and a healthy gut [25]. The experiment demonstrated that VIP effectively alleviated the magnitude of the mucosal damage. However, no significant differences were observed among treatments in VCR. Moreover, enteric pathogen and endotoxin translocations are known to increase paracellular permeability through tight junction (TJs) alterations [26]. Numerous studies using animals and cell cultures indicate that occludin plays crucial roles in the TJs structure and permeability in the intestinal epithelia [27]–[29]. In the study, administration with VIP alleviated ETEC K88 induced reduction of occludin expression in the ileum. Previous research also indicates that VIP has an important role in the nerve-mediated maintenance of intestinal barrier function by acting on ZO-1 [30]. Clarke et al. reported that invasive bacteria could lead to changes in TJ protein expression via TLRs-mediated pathways [31]. VIP protects the colonic epithelial barrier by minimizing bacterial-induced redistribution of tight junction proteins in part through actions on myosin light chain kinase (MLCK) and myosin light chain phosphorylation (p-MLC) [32].

Studies have shown that the function of the intestinal barrier may be regulated by a network of multiple cytokines, including ILs, IFNs and TNF-α [33]. An imbalance of pro-inflammatory cytokines and anti-inflammatory cytokines is another important mechanism of intestinal mucosal injury. The primary function of S-IgA is referred to as immune exclusion, a process that limits the access of numerous microorganisms and mucosal antigens to these thin and vulnerable mucosal barriers [34]. In the current study, the serum levels of S-IgA, IL-4, IL-10 and TGF-β from the ETEC K88 infected-pigs receiving VIP were higher than those from the ETEC K88 infected-pigs alone. It has been shown that VIP increase production of anti-inflammatory cytokines such as IL-10, TGF-β and IL-1Ra [12], [35]. TNF-α, IL-6 and IFN-γ play important roles in various inflammatory reactions and are highly correlated with the severity of inflammation. IFN-γ is the main Th1-type cytokine produced by T effector lymphocytes. Under pathophysiological conditions, pro-inflammatory cytokines, antigens, and pathogens contribute to barrier impairment [36], [37]. Moreover, weaning is associated with upregulation of IL-1, Il-6, and TNF-α in the intestine, and this early inflammatory response may contribute to both anatomical and functional intestinal disorders in piglets [38]. However, treatment with VIP significantly decreased the levels of TNF-α, IL-6 and INF-γ, suggesting that VIP may improve the permeability of the intestinal mucosa and protect the intestinal. The results are consistent with the report that VIP/PACAP protect mice from the lethal effect of high endotoxemia through the inhibition of TNF-α and IL-6 [39]. Modulatory effect of VIP on immune abnormalities mitigated the damage and inflammation of the intestinal immune response, which may also be one of the mechanisms VIP reducing rates of diarrhea in infected-piglets. Postnatal development of porcine IMIS was accompanied by a substantial increase in the secretion of neuropeptides/enzyme tested and that these molecules may participate in the functional maturation of immunoregulatory/bactericidal mechanisms of the local (intestinal) immune defense in young pigs [40]. Recent study indicated that inhaled VIP exerts immunoregulatory effects in sarcoidosis [41]. VIP and urocortin protect from the lethal effect of *E.coli* and cecal ligation and puncture (CLP)-induced sepsis and this protection is paralleled by a decrease in the systemic levels of high mobility group box 1 (HMGB1) [42]. The therapeutic effect of VIP was initially attributed to the down-regulation of a wide panel of inflammatory mediators and to the inhibition of autoreactive T_H_1 cells [43], [44]. VIP is now recognized as playing a major role in the regulation of Th1/Th2 balance [45]. Mounting evidence indicates that VIP via multiple mechanisms to counter inflammatory factors. VIP is also produced by lymphoid cells and exerts a wide variety of immunological functions, including control of homeostasis of the immune system by ligand-receptor signaling to immunocompetent cells, regulation of the production of anti- or pro-inflammatory mediators, changing of expression of co-stimulatory molecules leading to switching of Th1 to Th2 response, and stimulation of B cell differentiation and production of IgA antibodies [46], [47]. Anti-inflammatory effect of VIP is ascribed to its ability to abrogate phagocytosis and chemotaxis of macrophages [48] and to inhibit T cell proliferation and migration [49].

Intestinal microorganisms participate in various physiological functions, by which they influence their hosts. Enteric pathogens may cause several damages to intestinal cells, including interference in the epithelial cell signaling that controls both the transcellular and paracellular secretion pathways; concequently, protection against pathogenic and conditionally pathogenic microorganisms in the form of colonization resistance is most important [50]. In the current study, the microbial community of piglets in each treatment has been determined in detail. The obtained results indicated that ETEC K88 challenge diminished colon and caecum contents bacterial diversity and abundance. Administration with VIP increased these measurements, which may be associated with its capability of stimulating diverse microbial communities to colonize the GIT. An increase in microbial diversity has been associated with increased ecosystem stability and resistance to pathogen invasion [51]. The effect of VIP on intestinal bacterial community may be significant for its role in improving growth performance, reducing diarrhea ratio, and exerting immunoregulation function. Many researches demonstrate that the gut microflora plays an important role in the maintenance of animal health, improving immunity, participating in the absorption and metabolism of nutrients. Although the involved mechanisms remain unclear, similar to other neuropeptides [52], VIP shares some properties with antimicrobial peptides, such as small size, cationic charge, and amphipathic design. In addition, VIP is abundantly present in physical barriers of the body, physiological fluids, and immunoprivileged sites [53]. VIP is released under microbial-induced inflammation [54], [55]. *Bacteroidetes* and *Firmicutes* were prevalent members of the intestinal bacterial communities in each treatment. These results were consistent with a previous report. The gut microbiota of pigs mainly consists of the *Bacteroidetes* and *Firmicutes* divisions [56], just as mice and humans. In the study, ETEC K88 challenge increased abundance of *Bacteroidetes* in colon content, increased the relative proportion of *Prevotellaceae_uncultured* in colon and caecum content compared with the control group. The proportion of *Bacteroidetes* had a negative correlation with the body weight [57]. Interesting, although administration of VIP alone did not change the intestinal morphology and the mucosal immune function, it could reshape the community structure in colon and caecum content. The possible mechanism of VIP affecting the gut microbial community structure may be associated with antimicrobial activity against certain groups of microorganisms and its secretory effects on intestinal epithelia. VIP displayed antimicrobial activity against *Escherichia coli* ATCC25922 and *Pseudomonas aeruginosa* ATCC 27853 in vitro [15]. Two VIP derivatives kill various non-pathogenic and pathogenic Gram-positive and Gram-negative bacteria through a mechanism that depends on the interaction with certain components of the microbial surface, the formation of pores, and the disruption of the surface membrane [16]. VIP binds to crypt cell receptors and triggers secretion of NaCl and water [58], [59]. VIP strongly potentiated carbachol-induced mucin secretin [60]. Mourad and Nassar (2000) showed that VIP plays a role in heat labile enterotoxin (LT) and heat stable enterotoxin type A (STa) induced intestinal secretion and may be the final putative neurotransmitter in the pathophysiology of these toxins [61]. The potential advantages of the secretory effect for the host can be the flushing of the intestinal lumen and the clearing of pathogenic microbes. In pigs, the microbial ecosystem undergoes massive fluctuations in the time after weaning, and pigs are prone to enteric dysbiosis until a stable autochthonous microbiota has been developed [62]. The impact of bacteria on intestinal barrier function is clearly illustrated by the action of specific pathogenic enteric bacteria that have evolved remarkable means to penetrate and circumvent this important host defense mechanism [63]. Some bacteria such as *Lactobacillus plantarum* appear to modulate the epithelial barrier through the action of secreted protein (LGG p40) whereas other such as *Clostridium* likely influence the barrier through production of metabolites (SCFA). In view of the richness and diversity of the microbiota, it would be important to identify microorganisms with barrier protective function. Understanding the intricate relationship between epithelial barrier, microbe, and VIP would undeniably contribute key knowledge that could be harness for therapeutic purpose.

TLRs are one of the most important pattern recognition receptors (PRR) in innate immunity [64] and play a critical role in pathogen recognition and host defense [65], [66]. However, inappropriate TLR signaling can contribute to loss of tolerance and result in tissue injury, the best example of such injury is the intestinal damage ediated by the inflammatory response triggered by the interaction between lipopolysaccharide (LPS) and TLR4. LPS present in the outer membranes of some Gram-negative pathogens such as ETEC triggers the production of proinflammatory mediators that may contribute to intestinal inflammation and damage during the infection [67]. The modulation of TLR expression is one of the most recent functions in immunity attribute to VIP [68]–[70]. The current results showed that ETEC K88 infection increased the expression of TLR2, TLR4 and MyD88, and induced the phosphorylation of IKBα), p-ERK, p-JNK and p38. The best known signaling pathway activated by TLRs is associated with the MyD88 adapter. Key points in this pathway are TRAF6 activation by ubiquitination, IKK phosphorylation and activation of NF-κB and MAPK. Actually, many genes induced by TLR activation are controlled by both NF-κB and activator protein1 (AP-1). However, VIP treatment decreased the expression of TLR2, TLR4 and MyD88, and inhibited NF-κB and MAPK activation. Moreover, the activation of TLRs/NF-κB and TLRs/MAPK signaling was consistent with changes in the serum levels of IL-2, Il-6, IL-12p40, IFN-γ, and TNF-α. NF-κB is an essential transcription factor that regulates transcription of genes involved in the early inflammatory responses such as cytokines, chemokines and adhesion molecules and plays a central role in the pathobiology of inflammation [71]. Once activated, MAPK can phosphorylate transcription factors or transcriptional co-regulators or phosphorylate downstream kinases that induce expression of inflammatory mediators by extracellular stimuli [72]. The expression of proinflammatory genes such as TNF-α, IL-6 and IL-12 are induced via activation of NF-κB and MAPKs [73]. TGF-β is an anti-inflammatory cytokine produced in response to LPS stimulation that can attenuate the TLR-induced inflammatory response. Moreover, TGF-β is reported to produce ubiquitination and degradation of MyD88 protein, leading to a downregulation of MyD88-dependent activation of NF-κB and TNF-α production [74]. The present results suggested that modulatory effect of VIP on intestinal mucosal immunity may be by inhibition of TLR2/4-MyD88/NF-κB, and the inhibition of TLR2/4-MyD88/MAPK pathway. The negative effects of VIP signaling on NF-κB activation have been well described in the mouse. In *vivo* VIP treatment in the collagen-induced arthritis model prevents NF-κB nuclear translocation through the inhibition of IκB-α phosphorylation and degradation [75]. VIP downregulates the activity of several transduction pathways and their associated transcription factors essential for the transcriptional activation of most inflammatory cytokines, chemokines and costimulatory factors, including NF-κB, MAPK, interferon regulatory factor 1 (IRF1) and AP1 [76], [77]. As a small and hydrophilic molecule, VIP possesses excellent permeability properties that permit rapid access to the site of inflammation. Its high-affinity binding to specific receptors makes VIP very potent in exerting its immunomodulating and anti-inflammatory activities [78]. Although the exact mechanism of VIP-induced interference in TLRs expression and function in weaned piglets infected by ETEC K88 remains to be elucidated, emerging evidence suggests that the use of this neuroimmunopeptide represents one of the most promising future strategies for combating infections.

## Conclusion

The current study contributes to an understanding of the mechanisms through which VIP may benefit piglets during ETEC challenge. In general, these results confirmed that exogenous neuropeptide VIP improved the intestinal mucosal immunity and microbial community of weaning piglet after an oral challenge with ETEC (K88). The TLR2/4-MyD88 mediated NF-κB and MAPK signaling pathway may be critical to the mechanism underlying the modulatory effect of VIP on intestinal mucosal immune function and bacterial community. Although further studies are needed to evaluate the mechanisms involved, our observation supports the hypothesis that VIP down-mediated multiple proinflammatory pathways, including TLR-mediated pathways. Hence, such potentials of these molecular elements of porcine IMIS should be also monitored when an exogenous immunomodulation is applied to enhance defense of intestinal mucosal surface of weaned pigs against enteric pathogens. The research provides new insight into its use in the animal husbandry.
